# Oncogenic Viruses and Breast Cancer: Mouse Mammary Tumor Virus (MMTV), Bovine Leukemia Virus (BLV), Human Papilloma Virus (HPV), and Epstein–Barr Virus (EBV)

**DOI:** 10.3389/fonc.2018.00001

**Published:** 2018-01-22

**Authors:** James S. Lawson, Brian Salmons, Wendy K. Glenn

**Affiliations:** ^1^School of Biotechnology and Biomolecular Sciences, University of New South Wales, Sydney, NSW, Australia; ^2^Austrianova, Synapse, Biopolis, Singapore

**Keywords:** breast cancer, oncogenic viruses, mouse mammary tumour virus, bovine leukemia virus, human papilloma virus, Epstein–Barr virus, multiple viral infections

## Abstract

**Background:**

Although the risk factors for breast cancer are well established, namely female gender, early menarche and late menopause plus the protective influence of early pregnancy, the underlying causes of breast cancer remain unknown. The development of substantial recent evidence indicates that a handful of viruses may have a role in breast cancer. These viruses are mouse mammary tumor virus (MMTV), bovine leukemia virus (BLV), human papilloma viruses (HPVs), and Epstein–Barr virus (EBV-also known as human herpes virus type 4). Each of these viruses has documented oncogenic potential. The aim of this review is to inform the scientific and general community about this recent evidence.

**The evidence:**

MMTV and human breast cancer—the evidence is detailed and comprehensive but cannot be regarded as conclusive. BLV and human breast cancer—the evidence is limited. However, in view of the emerging information about BLV in human breast cancer, it is prudent to encourage the elimination of BLV in cattle, particularly in the dairy industry. HPVs and breast cancer—the evidence is substantial but not conclusive. The availability of effective preventive vaccines is a major advantage and their use should be encouraged. EBV and breast cancer—the evidence is also substantial but not conclusive. Currently, there are no practical means of either prevention or treatment. Although there is evidence of genetic predisposition, and cancer in general is a culmination of events, there is no evidence that inherited genetic traits are causal.

**Conclusion:**

The influence of oncogenic viruses is currently the major plausible hypothesis for a direct cause of human breast cancer.

## Aim of This Review

Although the risk factors for breast cancer are well established, namely female gender, early menarche and late menopause plus the protective influence of early pregnancy, the underlying causes of breast cancer remain unknown. The development of substantial recent evidence indicates that a handful of viruses may have a role in breast cancer. These viruses are mouse mammary tumor virus (MMTV), bovine leukemia virus (BLV), human papilloma viruses (HPVs), and Epstein–Barr virus (EBV—also known as human herpes virus type 4). Each of these viruses has documented oncogenic potential.

The aim of this review is to inform the scientific and general community about this recent evidence.

It is helpful to consider this evidence in the context of past studies. It is also helpful to consider the potential role of these four oncoviruses in a single review because there is emerging evidence that one or more of them may collaborate.

## Introduction

We have used an extension of the classic Hill criteria to review the evidence (1). These criteria are (i) strength of association (the odds ratio with the higher the strength of association between a causal agent the more likely an association), (ii) consistency (the confirmation of findings in repeated studies conducted by different researchers in different locations and at different times), (iii) specificity (the more specific the relationship between a causal agent and a disease the more probable there is an association), (iv) temporality (the time sequence where an event such as infection with a causal agent precedes a specific disease), (v) biological gradient (response to different concentrations or length of exposure to the causal agent) (vi) plausibility (conclusions drawn from previous evidence such as the oncogenic capacity of an infectious agent), (vii) coherence (overall the evidence is consistent), (viii) experimental evidence (the most valuable is a demonstration that the disease will not develop if the presumed cause is removed, such as use of an effective preventive vaccine), and (ix) analogy (an example is the proven cause of cancer by viruses in animals which may have parallels in humans). With respect to viruses, additional causal criteria to the Hill criteria are (i) viral genetic material is present in cancer tissues but rarely in normal tissues, (ii) the virus is capable of transforming normal cells into malignant cells, (iii) the viral oncogenic mechanism is understood, and (iv) the means of viral transmission has been identified. There are differences in the importance of each criterion. With respect to viruses and cancer the identification of viral genetic material and a significant odds ratio between cancer and normal tissues are of special importance.

The development of conclusive evidence for a viral cause of breast cancer is extremely difficult because the proportion of viral nucleic acids in a cancer sample is usually very small when compared with host-derived genetic material. MMTV and BLV are retroviruses. Retroviral genomes rarely exceed 10–12 kb, and hence constitute a minor fraction of the genome of the infected host cell. The infected cell type may constitute only a small fraction of the sample, and the infected cells may contain a relatively low number of viral genome copies (2). HPVs and EBV are DNA viruses. The viral load of high risk for cancer HPVs is 2,000-fold less in breast cancer when compared with cervical cancer (3). Although these viruses can be detected by whole-genome sequencing, amplification techniques such as nested polymerase chain reaction (PCR) can be required for their successful identification. However, because of these problems the outcomes of PCR can be inconsistent and even falsely negative. It can also be argued that these viral loads are so low that they may not be oncogenic.

### Techniques Used to Implicate Viruses in Breast Cancer

The techniques used for the identification of viruses in breast and other cancers are of fundamental importance to the validity of studies aimed at determining viral causes of cancer. These techniques include (i) various types of PCR—standard PCR which is based on extracts of DNA from target tissues and cannot be used to identify the location of viral gene sequences in specific cell types, and *in situ* PCR which can identify viral gene sequences in specific cells, (ii) *in situ* hybridization which can identify viral gene sequences in specific cells but which is much less sensitive than PCR amplification techniques, (iii) immunohistochemistry which can be used to identify viral proteins, and (iv) massive genome sequencing which can be used to identify specific viral gene segments but does not appear to be as sensitive as PCR. Because of its sensitivity, PCR remains as the most commonly used technique but is notoriously subject to contamination which can lead to false positive results. Joshi and Buehring have reviewed the techniques that have been used in studies of MMTV, HPVs and EBVs in breast cancer (4). They have shown there is considerable variation in the quality of these studies and have suggested a number of criteria which should be used to assess the validity of results. These criteria include (i) the use of *in situ* molecular methods (*in situ* PCR) either initially or as confirmation of results based on standard PCR so as to identify the cell type positive for the virus and to avoid contamination, (ii) the use of *in situ* hybridization tests, and (iii) the use of normal control breast tissues from women with no history of breast cancer and not from normal tissues adjacent to breast tumors.

In this review, we have used published meta-analyses as one basis for the selection of studies listed in the tables. While we did not identify any biases in these meta-analyses, we have only included in the tables studies which report results which include adequate controls and which meet the criteria outlined above.

## Mouse Mammary Tumor Virus

Some background is helpful to place the evidence in context. While working at the Jackson Laboratory in Maine in 1936, John Bittner discovered a cancerous agent, which he called a “milk factor,” which could be transmitted by milk from mouse mothers with mammary tumors to their mouse pups, who as adults, later developed mammary tumors (5). Graff et al., working in New York in the post—World War II years, identified viral particles in mouse milk which they showed could cause mammary cancer when injected intraperitoneally into laboratory strains of mice (6). In 1966, Peter Duesberg and Phyllis Blair, working at the University of California at Berkeley, identified this virus as a retroviral RNA virus, similar to other RNA tumor viruses and which has become known as MMTV (7). The biology of MMTV has been intensively studied and is the proven cause of mammary cancers in wild (feral) and experimental mice (8). MMTV has been identified in a number of different organs in the mouse including the mammary glands, the prostate, salivary glands, and the lymphatic system (8). MMTV has also been identified in several different mammal species including humans, dogs, cats, and monkeys (9).

Mouse mammary tumor virus became an obvious suspect as a causal agent in human breast cancer and financial resources from the President Richard Nixon war on cancer were allocated to research in this field. A major breakthrough came with the identification of RNA in human breast cancer that was homologous to MMTV RNA (10). This observation was confirmed during the next decade at the DNA level (11–13). However, it was difficult to distinguish MMTV gene sequences from those of the human endogenous retrovirus (HERV). HERV gene sequences are very similar to MMTV and may be the remnants of MMTV viruses that have become integrated into the human genome over millennia (14). This problem was overcome by the Beatriz Pogo group working in the Mount Sinai Medical Center in New York, by their identification of MMTV envelope gene sequences which were unique to MMTV (15). Their work lead to a resurgence of interest in MMTV and human breast cancer. The likely lifecycle of MMTV in humans appears to be very similar to the lifecycle of MMTV in mice. This offers an almost unique animal model which has allowed investigators to follow specific lines of enquiry.

### Identification of MMTV in Human Breast Cancer

Using hybridization with cloned MMTV DNA techniques (16–18), Callahan et al., Westley and May, and May and Westley, identified related sequences in human breast cancer cells. Using similar hybridization methods, Szakacs and Moscinski (13) identified the entire provirus of MMTV with a typical structure of a retrovirus [long terminal repeat (*LTR*), group specific antigen (*gag*), capsid region, polymerase (*pol*), reverse transcription region, and envelope (*env*) in 7 (13%) of 52 human breast cancers]. The identification of MMTV sequences by hybridization techniques in these studies is important because of the later controversies related to the use of PCR techniques.

With respect to the review of MMTV in human breast cancer, the publications included in a meta-analysis by Wang et al. supplemented by more recent publications were considered and included in Table 1 (19). Only case control studies with adequate controls are included. The criteria for inclusion in this meta-analysis were (i) studies had to use PCR-based techniques to detect regions of the MMTV envelope gene that have low homology to known HERVs in tissues and (ii) only studies on breast cancer in females. The results indicate there is a 15-fold higher prevalence of MMTV *env* sequences in human breast cancers when compared with non-cancer controls. It should be noted that these studies are heterogeneous and accordingly this meta-analysis cannot be regarded as definitive. Despite this reservation, MMTV *env* gene sequences were identified in breast cancers but not normal breast controls by *in situ* PCR in three of the studies in Table 1 (20–22). Several studies which did not identify MMTV gene sequences have been omitted from Table 1 because of inadequate methods as judged by the criteria of Joshi and Buehring (4).

**Table 1:** Identification of MMTV sequences in breast cancer and comparison non-cancer breast specimens (case–control studies).

Reference	Location	Diagnosis	Non-cancer controls	Specimen identification technique	MMTV positive/total cancer specimens	MMTV positive/total non-cancer specimens
Wang et al. (15)	US	Invasive	Normal breast	Frozen PCR	121/314 (38.5%)	2/107 (2%)
				ISH		

Etkind et al. (23)	US	Invasive	Normal breast	Frozen PCR	27/73 (37%)	0/35 (0%)
				ISH		

Melana et al. (24)	US	Invasive	Adjacent normal breast	Formalin PCR	32/106 (30%)	1/106 (1%)
				ISH		

Melana et al. (25)	Argentina	Invasive		Frozen PCR	23/74 (31%)	1/10 (10%)

Ford et al. (26)	Australia	DCIS	Benign	Formalin PCR	DCIS 5/19 (26%)	2/111 (2%)
		Invasive			IDC 14/26 (54%)	

Ford et al. (20)	Australia	Invasive	Normal	Formalin. *in situ* PCR	45/144 (31%)	0/20 (0%)
		DCIS		ISH	2/8 (25%)	

Zammarchi et al. (27)	Italy	Invasive	Adjacent normal breast	Frozen PCR	13/43 (30%)	1/8 (12.5%)
				Microdissection		

Bindra et al. (28)	Sweden	Invasive	Adjacent normal breast	Frozen PCR	0/18 (0%)	0/11 (0%)

Hachana et al. (29)	Tunisia	Invasive	Adjacent normal breast	Frozen PCR	17/122 (14%)	0/122 (0%)

Mazzanti et al. (21)	Italy	DCIS	Normal cosmetic	Formalin PCR, *in situ* PCR, ISH	40/49 (82%)	0/20 (0%)
		Invasive			7/20 (35%)	

Lawson et al. (30)	Australia	DCIS	Normal cosmetic	Formalin PCR, *in situ* PCR	33/74 (45%)	0/29 (0%)
		Invasive				

Glenn et al. (22)	Australia	Invasive	Normal cosmetic	Frozen PCR: *in situ* PCR	39/50 (78%)	13/40 (33%)

Slaoui et al. (31)	Morocco	Invasive	Adjacent normal breast	Formalin PCR	24/57 (42%)	6/18 (33%)

Cedro-Tanda et al. (32)	Mexico	Invasive	Adjacent normal breast	Frozen	57/458 (12%)	72/458 (16%)
				PCR		

Reza et al. (33)	Iran	Invasive	Adjacent normal breast	Formalin	12/100 (12%)	0/100 (0%)
		DCIS		PCR		

Naushad et al. (34)	Pakistan	Invasive	Normal	Formalin PCR	83/250 (29%)	0/15 (0%)

Shariatpanahi et al. (35)	Iran	Invasive	Benign	Formalin PCR	19/59 (32%)	3/59 (5%)

*DCIS, ductal carcinoma in situ; PCR, polymerase chain reaction; ISH, in situ hybridization; MMTV, mouse mammary tumor virus*.

There are geographical differences in the prevalence of MMTV in mice which may influence the prevalence of MMTV in humans (36). The higher the prevalence of one species of house mouse, Mus domesticus, the higher the prevalence of human breast cancer (36). The prevalence of MMTV positive breast cancers in Western countries is broadly consistent between 30 and 40% and in Asian countries between 10 and 20% (36). These differences may, in part, be due to the differences in exposure to MMTV in mice (36).

There are wide variations in the identification of MMTV in breast cancers from the same countries. For example, the identification of MMTV in breast cancers in Mexico varied from nil to 16% and in Iran nil to 12%. By comparison the outcomes of multiple studies in the US, Italy, and Australia are broadly similar. It is likely that some of these differences are due to the technical difficulty of identifying MMTV sequences by PCR. We have recently shown that the outcomes of PCR used to identify MMTV can differ despite the use of the same specimens (37). However, PCR is the most sensitive technique available for the detection of low level viral infections and the outcomes should not be discounted because of these difficulties. In some of the published studies the methods are clearly inadequate as for example the omission of a positive control or the failure to achieve a positive control outcome (38–40). These studies have not been included in Table 1.

It should be noted that hybridization techniques have also been used to successfully confirm the outcome of PCR amplification techniques in 5 of the 17 studies in Table 1. *In situ* PCR techniques were also used which confirmed the outcomes of standard PCR in two studies. *In situ* PCR is less likely to give false negative and positive outcomes. It should also be noted that the identification of MMTV were similar whether PCR primers based on envelope gene or LTR gene MMTV sequences are used (41). MMTV encodes a characteristic dUTPase in the gag gene and future confirmatory studies using primers in this region might be useful (42).

Overall, the much higher prevalence of MMTV positive identification in breast cancers when compared with non-cancerous breast tissues is suggestive of a role for MMTV in a subset of human breast cancers.

### Whole-Genome Sequencing

The identity of MMTV in human breast cancers has been confirmed by whole-genome sequencing (43, 44). However, MMTV sequences were identified in only 3 of over 800 breast cancer specimens from The Cancer Genome Atlas (TCGA). This is an indication that PCR is much more sensitive than whole-genome sequencing (2).

The almost complete genome of MMTV in human breast cancers has been identified with 84–99% homology to MMTV in mouse breast cancers (45, 46). Breast cancer cells produce MMTV-like viral particles that are similar to the mouse virus with sequence homologies 85–95% the same as MMTV (45). Nucleotides, gene sequences and number of base pairs in the MMTV genome are virtually identical in both mouse and human breast cancers (45–47).

### Specificity

Mouse mammary tumor virus is unlikely to be uniquely specific to breast cancer as it has been identified in several additional cancers including lymphoma, prostate, liver, and endometrial cancer (48–50). There is no evidence to determine whether MMTV is causal in these cancers.

### Breast Cancer-Related Gene Expression

The same cancer-related genes are deregulated in both MMTV-associated mouse and human breast cancer (51, 52). These genes are mainly associated with cell proliferation.

### MMTV Proteins in Human Breast Cancer

The MMTV envelope proteins (gp 36 and gp 52) have been identified in primary cultures of human breast cancer cells which confirm earlier findings (53–55).

### MMTV Biological Gradient

The MMTV viral load appears to increase as human breast cancer progresses but falls in late stage invasive breast cancer (20, 21). In a study based on Italian women, MMTV was not identified in normal breast tissues from cosmetic breast surgery, but MMTV was identified in increasing prevalence in 27% of breast hyperplasias and 80% of non-invasive ductal carcinoma *in situ* but less prevalent in invasive ductal carcinomas which were 35% MMTV positive (21).

### Serology

Serological-based studies of MMTV in human breast cancer have produced conflicting outcomes. Older studies identified serological responses to MMTV (56–58). Indeed, Dion et al. reported that laboratory workers exposed to MMTV showed serological responses to the MMTV *env* (gp52 and gp34) and gag (p12, p18, p28) proteins (58). However, later studies based on more modern methods did not achieve clear outcomes (59). Although 30% of the sera from women with breast cancer showed some serological reactivity against MMTV virus, none could be immunoprecipitated using purified MMTV proteins (59).

### Temporality: MMTV Infection and Subsequent Breast Cancer Time Sequence

Five of six Australian women with MMTV positive benign breast biopsy tissues 1–11 years later developed MMTV positive breast cancer (37). The prior benign and later breast cancer specimens were from the same individual patients (37).

### Superantigen (SAg) Expression

Mouse mammary tumor virus SAgs play an essential role in MMTV-associated mouse mammary tumors (60). MMTV infection of antigen presenting cells such as B lymphocytes and dendritic cells leads to the expression of virus SAgs which in turn stimulate T cells to produce cytokines that encourage the proliferation of infected B lymphocytes. MMTV is then conducted throughout the body by these lymphocytes which facilitate the entry of MMTV into its target organ (the breast or other organs). MMTV SAg is highly expressed in MMTV-associated human breast cancer and may have a similar role in humans as it does in the mouse (61). Human T cells respond to MMTV SAg (62).

### Hormone Response

Mammary tumors in mice are hormone dependent (63–65). In humans, MMTV hormonal response elements in breast cancers appear to promote cell growth as they do in the mouse system (66).

### MMTV Capacity to Infect Human Breast Epithelial Cells

Mouse mammary tumor virus is capable of infecting human breast cells in culture and can randomly integrate its genomic information into the genome of the infected cells (67–69). When MMTV is integrated into the human genome the flanking sequences are of human and not mouse origin which is an indication of an exogenous infection rather than contamination (70). Integration of MMTV into the human genome appears to be random and in multiple locations as has been previously observed in mice (70).

Etkind et al. (71) identified a family in which the father, mother and daughter living together, all developed breast cancer. Each of the three breast cancers contained *env* gene sequences that were at least 98% homologous to the MMTV *env* sequences found in laboratory mouse strains. This provides support for an infective virus transmission.

### MMTV Transmission

Mouse mammary tumor virus has been identified in human milk from normal lactating mothers (72). The prevalence of MMTV in human milk is significantly higher among women who are at greater than normal risk of breast cancer (73). MMTV gene sequences have been identified in human saliva (74). MMTV gene sequences are present in saliva in 27% of normal children, 11% of normal adults and 57% of women with breast cancer (74). This suggests saliva as a route in inter-human infection. MMTV gene sequences have been identified in dogs and cats (9, 75). Women with companion dogs are at twice the expected risk of breast cancer (76). In addition the biological and histological characteristics of canine mammary tumors are very similar to human breast cancer (77). These observations are suggestive of transmission of MMTV in dog saliva to humans.

### Identification of MMTV in Other Animals

Mouse mammary tumor virus is the proven cause of mammary cancers in wild (feral) mice (8). MMTV-like gene sequences have been identified in 20% of mammary tumors in dogs and 33% of cats (75). Gene sequences with high homology to MMTV have been identified in rhesus monkeys and cats although the tissues studied were not mammary tumors (9).

### Histopathology

The histopathology in the early stages of MMTV-associated mouse and human breast cancers are very similar and in some cancers are virtually identical (30, 78). However, these characteristics are not sufficiently different from several other cancers in humans, such as basal cell skin carcinomas, to act as diagnostic criteria. The histological characteristics of breast cancer in wild and experimental mice were studied in detail by Thelma Dunn during and after the 1940s (79). Over 90% of mouse mammary tumors in wild (feral) mice are adenocarcinomas. Dunn classified these tumors as types A and B. The breast glands are present in type A. Type B, the most common type, is characterized by dense round cancer cells without glands.

Wellings made the first observation that some human breast cancer specimens had similar histopathology characteristics to MMTV-associated mouse breast cancers (80). However, there is a belief that MMTV-associated mouse breast cancers do not resemble human breast cancers. This belief may be based on the observations made by Hamilton in 1974 (81). At that time most observations were made at a late stage in the development of breast cancer. Hamilton described human invasive ductal breast carcinomas which were characterized by streams of elongated malignant cells surrounded by dense connective tissues which were clearly different from the histopathology of mouse breast cancers. This may explain Hamilton’s conclusions as Wellings observations were based on the early proliferative stages of mouse breast and human breast cancers whereas Hamilton’s conclusions were made on the late invasive stage of human breast cancer (80, 81). The histopathology of early proliferative stages of human breast cancer is very different from late stage human breast cancer.

### Mechanisms of MMTV Oncogenesis in Human Breast Cancer

Even though MMTV has been classified as a non-acutely transforming retrovirus, there is experimental evidence that proteins expressed by the MMTV envelope gene are capable of malignantly transforming normal human breast epithelial cells (82). However, the mechanisms by which MMTV may cause cancer are far from clear. It has been experimentally demonstrated that integration of MMTV proviral DNA into the target genome near one or more of the proto-oncogenes such as Wnt-1 and Fgf are associated with the development of mouse mammary tumors (83). However, integrations in the vicinity of multiple proto-oncogenes seem to be required, together with other genetic insults as well as predisposing genetic mutations (84). The insertion of the MMTV enhancers in the vicinity of proto-oncogenes results in their deregulated expression which stimulates cell growth (84). It is of interest that Wnt-1 expression is higher in specimens of MMTV *env*-positive ductal carcinoma *in situ* and invasive ductal carcinoma, than in MMTV *env*-negative specimens (30). However, if MMTV is causing human breast cancer like it does in mice, then MMTV DNA should be readily detectable in tumors since they are derived by clonal outgrowth of one (or a few) originally infected cells that carry an integrated provirus near a cellular oncogene. Moreover, most MMTV-induced mammary tumors seem to contain 10 or more proviral integrations and it is thought that MMTV-induced tumors arise when these multiple integrations occur in a single cell (85). However, the relatively low levels of MMTV that are detected in human breast cancers suggest that the virus is affecting oncogenesis by mechanisms other than enhancer insertion.

Mouse mammary tumor virus envelope proteins have been proposed to be involved in tumorigenesis (86, 87). MMTV envelope protein overexpression can lead to morphological changes in normal 3-dimensional mouse breast cell cultures (86). As noted previously, the envelope proteins of Jaagsiekte Sheep Retrovirus which like MMTV is a beta retrovirus, can directly transform cells (88).

Mouse mammary tumor virus-encoded proteins (such as Rem, Sag, Naf) or as yet uncharacterized proteins analogous to those of other complex retroviruses such as Tax may also have a role in breast cancer (87). Further, it has been shown that MMTV, like some other retroviruses, can influence infected host cell miRNA, enhancing the expression of members of the oncogenic mi RNA cluster, mi-17–92 (89). There is also the intriguing possibility that MMTV and HERVs may interact and thus also play a role (86).

It is also conceivable that MMTV infection activates a latent human DNA virus such as EBV or human papillomavirus (HPV)—both of which are candidate viruses associated with human breast cancer, in an analogous fashion to the way that human immunodeficiency virus has an indirect role in Kaposi’s sarcoma (87).

Apolipoprotein B editing complex (APOBEC) family genes encode deaminase enzymes that edit DNA and/or RNA sequences thus playing a central role in the control of virus infections, including retroviruses (90). APOBEC enzymes normally function in innate immune responses, including those that target retroviruses, suggesting links between mutagenesis, immunity and viral infection in cancer development. In mouse models, APOBEC3 has been shown to inhibit MMTV infections and viral replication and to restrict milk borne MMTV virions (90–92). Abnormal expression of APOBEC3B may reduce its protective effects against MMTV. Inactivating mutations and deletions in APOBEC3B are also thought to play a role in breast cancer development. Aberrant expression of APOBEC3B has also been shown to be induced by DNA viruses such as HPV, specifically in breast cancer (93, 94). Moreover, a deletion polymorphism in the APOBEC3B gene cluster on chromosome 22 is associated with elevated breast cancer risk and a specific APOBEC mutation pattern has been described in a number of cancers, including breast cancer, suggesting a functional link with cancer development (95, 96). This mutation pattern is linked to APOBEC3B expression specifically in breast cancer and leads to DNA damage that could select TP53 inactivation, thereby resulting in tumor heterogeneity (97). Recently, it has been shown that there is a significant increase in APOBEC-mediated mutagenesis in HER+/HER2 metastatic breast tumors when compared with early stage primary breast cancer (98). Taken together, these studies suggest that the APOBEC family and particularly APOBEC3B may play a central role in the early stages of breast cancer induction. Additional studies are required to demonstrate a link between MMTV, APOBEC, and breast cancer.

### Analogy between MMTV-Associated Human Breast Cancer and MMTV-Associated Mouse Mammary Tumors

The biology of MMTV in humans appears to be very similar to MMTV in mice (8). The evidence related to MMTV-associated human breast cancer and MMTV induced mouse mammary tumors, is remarkably similar. The key evidence is summarized in Table 2.

**Table 2:** Comparative evidence.

Mouse mammary tumor virus (MMTV)	Mouse mammary tumors	Human breast cancer
MMTV nucleotide and gene sequences	Complete MMTV genome of 9,900 base pairs identified (99–101)	Almost complete MMTV genome identified with 84–99% homology with mouse MMTV genome (45, 46). MMTV identification confirmed by whole-genome sequencing (45)

MMTV virus particles	MMTV virus particles visualized in mouse milk (99)	MMTV virus particles visualized in human milk (99)

MMTV breast cancer prevalence	MMTV identified globally in mouse mammary tumors (36)	MMTV-like virus identified globally in breast cancers (36)

Cancer-related gene expression	Same cancer genes deregulated in mouse mammary tumors and human breast cancer (51, 52)	Same cancer genes deregulated in mouse mammary tumors and human breast cancer (51, 52)

Temporality—MMTV infection time sequence	MMTV present in 0–50% of wild mice. 1% develop breast cancer (102, 103)	MMTV present in prior benign breast later breast cancer (37)

MMTV protein expression	MMTV proteins expressed (8, 104)	MMTV proteins expressed (53)

MMTV superantigen expression (SAg)	MMTV SAg plays an essential role in MMTV mouse breast cancer (60)	Human T cells respond to MMTV SAg (61). MMTV-like SAg identified in human breast cancer (61)

MMTV serum antibodies	Positive serological response to MMTV (105)	Positive serological response to MMTV (56–58). Serology not confirmed (59)

Hormone responsiveness	Mouse mammary tumors hormone dependent (8)	MMTV hormonal response elements promote cell growth (66)

MMTV transmission	MMTV transmitted by mouse milk to pups, which develop mouse mammary tumors as adult mice (8). MMTV present in mouse salivary gland—possible route of transfer (106)	Potential transmission by human milk and human saliva (72–74)

Infection experiments	Exogenous MMTVs target dendritic cells and B lymphocytes in intestinal lymphocytes and mouse mammary cells (8)	MMTV infects intestinal lymphocytes, human breast cells and randomly integrates into human genome (67, 68, 70, 107)

MMTV morphology	Classical MMTV mammary tumor morphology—Dunn types A and B (79)	Some MMTV positive breast cancers similar to MMTV positive mouse mammary tumors (30, 78)

*MMTV-associated human breast cancer and MMTV-associated mouse mammary tumors*.

In mice MMTV is spread by the milk of infected mouse mothers and is acquired by suckling pups (8).

Ingested MMTV enters T and B cells located in Peyer’s patches of the gastrointestinal tract of infected mouse pups. This spread of MMTV infection requires activation of T and B lymphocytes by the viral Sag. Sag activation allows the virus to amplify in lymphocytes prior to transmission of the virus to mammary epithelial cells. Ultimately the virus is transported by infected lymphocytes to the mammary glands where oncogenic transformation takes place (8).

In humans, MMTV infects lymphocytes located in the intestine and randomly infects and integrates into the human genome located in normal breast epithelial cells (67–70, 107).

## Bovine Leukemia Virus and Breast Cancer

Bovine leukemia virus is a delta retrovirus which is closely related to the human T cell leukemia virus 1 (108). It is the cause of leukemia in beef and dairy cattle. BLV has typical retroviral genome regions: *LTR* (promoter region), *gag* (group specific antigen, capsid region), *pol* (polymerase, reverse transcription region, which synthesizes a DNA copy of the BLV RNA genome), and *env* (envelope). However, unlike other oncogenic retroviruses, delta retroviruses have an additional region, *tax* (trans-activating region of the X gene), which has regulatory functions and is oncogenic to host cells. *Tax* causes malignant transformation by inhibition of DNA repair and disruption of cellular growth control mechanisms.

Clinical leukemia develops in less than 5% of infected cattle, however, BLV-infected lymphocytes are also found in the blood and milk of sub clinically infected cows (108). BLV-infected cattle herds are found worldwide. In the USA, approximately 38% of beef herds, 84% of all dairy herds, and 100% of large-scale dairy operation herds are infected (109). In Argentina, which has a major cow’s milk and beef industry, a majority of the herds are infected with BLV (110). In contrast, because of the culling of infected cattle, BLV infections have been virtually eliminated in Australian and European cows (111). However, virtually all Australians have consumed cow’s milk or milk-based products such as ice cream and cheese on a regular basis since infancy and many would have been exposed to BLV-infected milk. It is possible that any oncogenic influences of BLV may take decades to develop into breast cancer and accordingly no reduction in breast cancer prevalence can be anticipated for many years.

### Identification of BLV in Human Breast Tissues

Bovine leukemia virus gene sequences have been identified by PCR in human breast cancers (112–115). In a case control study based on PCR, 67 (59%) of 114 US breast cancers were positive for BLV when compared with 30 (29%) of 104 normal breast controls—odds ratio of 3 (114). There was a similar prevalence pattern in a recent study of breast cancer in a series of women from Texas (116). Of interest in this study was the increase in prevalence of BLV in benign breast tissues (19.6%), pre-malignant breast tissues (34%) and malignant breast tissues (57.4%) (116). In a case control study also based on PCR, 40 (80%) of 50 Australian breast cancers were BLV positive when compared with 19 (41%) of 46 normal controls—odds ratio 4.7 (115). There is an anecdotal report of BLV being able to infect human cells but these were of neural origin (117). The data in these studies are shown in Table 3.

**Table 3:** Bovine leukemia virus and human breast cancer.

Reference	Breast specimens	Diagnosis	Non-cancer controls	Identification technique	Bovine leukemia virus positive breast cancer (%)	Bovine leukemia virus positive non-cancer controls (%)
Giovanna et al. (112)	Columbia	Invasive breast cancers	Benign breast tissues	Standard PCR	19/53 (36%)	24/53 (45%)
Buehring et al. (114)	US	Invasive breast cancers	Normal breast tissues	*In situ* PCR	67/114 (59%)	30/104 (29%)
Buehring et al. (115)	Australia	Invasive breast cancers	Benign breast tissues	*In situ* PCR	40/50 (80%)	19/46 (41%)
Baltzell et al. (116)	US	Invasive breast cancers	Benign breast tissues	*In situ* PCR	35/61 (57%)	20/103 (20%)
Gillet et al. (118)	US	Invasive breast cancers	Normal next to breast cancer	Whole-genome sequencing	0/51	0/19

Bovine leukemia virus capsid proteins have been identified in the serum of 191 (74%) of 257 healthy human adults (119).

Contrary to these positive findings, Zhang et al. (120) did not identify BLV by PCR in Chinese breast cancers. However, the methods used do not appear to be adequate (121).

Using whole-genome sequencing methods BLV was not identified in 51 breast cancers (118). The reason for the negative outcome based on whole-genome sequencing is not clear. A plausible reason is that whole-genome sequencing techniques are not as sensitive as amplification techniques such as PCR and the authors were assuming that BLV would have to be present in all cells of the tumor and this may not be the case (2). BLV gene sequences were identified by PCR techniques in human breast cancer in the studies by Giavanni et al. (112) and Buehring et al. (113) which is an indication that their observations are sound.

### Temporality: BLV Infection and Subsequent Breast Cancer Time Sequence

Bovine leukemia virus was identified in 23 (74%) of 31 benign breast biopsy tissues 3–10 years before the development of BLV positive breast cancers in the same patients (115). The prior benign and later breast cancer specimens were from the same individual patients (115).

### Epidemiology

Bovine leukemia virus and BLV-infected cells are present in colostrum and milk of most infected cows (108). This is the most likely means of transmission from cows to humans. There is a striking geographical correlation between breast cancer mortality and milk and bovine meat consumption (122). Zur Hausen and de Villiers have demonstrated consistent correlations between the consumption of bovine meat and cow’s milk and the prevalence of breast cancer (122). Their data is based on comparisons between countries. Countries such as the US, the United Kingdom, Australia, and Germany each have high consumption of bovine meat and milk and high prevalence of breast cancer. Countries such as India, Japan, Korea, and China have low consumption of bovine meat and milk and low prevalence of breast cancer. The relevant data is shown in Table 4. Women with lactose intolerance and who have a low consumption of milk and dairy products, have a significantly lower risk of breast cancer than other women and other family members (123). In a Swedish based study by Ji et al., 22,788 individuals with lactose intolerance and who consumed only small quantities of cow’s milk and other dairy products, had a 20% lower prevalence of breast cancer when compared with 69,922 siblings and parents (123). Although this might indicate the involvement of a virus transmitted in milk or dairy products as hypothesized by zur Hausen and de Villiers (122), there are additional possible explanations. These include (i) caloric restriction which is associated with a lower incidence of breast cancers (124), (ii) the presence of growth factors in milk fats which may be associated with breast cancer risk (125), (iii) alterations to the human gut microbiome by avoidance of milk consumption and which may influence the development of cancers (126), and (iv) the potentially protective effects of dietary factors such as consumption of plant milk, including soy and rice milks, which are often consumed by women with lactose intolerance (127).

**Table 4:** Breast cancer mortality, incidence, milk, and bovine meat consumption high- and low-risk countries (per 100,000 women age adjusted) (2, 12, 128).

Country	Breast cancer mortality/100,000 women	Milk (kcal/person/day)	Bovine meat (kcal/person/day)
Argentina	19.9	187	347
United Kingdom	17.1	215	65
Germany	15.5	128	37
United States	14.9	197	115
Australia	14.0	186	126
Japan	9.8	84	28
South Korea	6.1	20	46

## High-Risk Human Papilloma Viruses and Breast Cancer

### Identification of High-Risk HPVs in Human Breast Tissues

High-risk HPVs are the cause of cervical cancer and have a role in head and neck and other cancers. The HPV viral load in breast cancer is extremely low when compared with HPV in cervical cancer (3). As a consequence, identification of HPV in breast tumors is difficult whether by amplification techniques such as PCR or whole-genome sequencing. The low viral load is the likely reason that several laboratories have not been able to detect HPV. On the other hand HPV gene sequences have been identified in breast tumors in over 40 studies conducted in 20 countries (129, 130). With respect to the review of HPVs in human breast cancer, the publications included in the meta-analyses by Bae et al. and Choi et al. supplemented by more recent publications have been considered and included in Table 5 (129, 130). These data indicate that high-risk HPVs are fourfold more prevalent in breast tumors when compared with non-cancer controls.

**Table 5:** Identification of high-risk HPVs in breast cancer and benign or normal breast controls in case control studies.

Reference	Country	Identification technique	HPV-positive breast cancers/total breast cancers	HPV-positive non cancer breast/total non-cancer breast controls	Main HPV types
Yu et al. (131)	Japan/China	PCR	18/52 (35%)	0/15 (0%)	18, 33
Damin et al. (132)	Brazil	PCR	25/101(25%)	0/41 (0%)	16, 18
Tsai et al. (133)	Taiwan	PCR	8/62 (13%)	2/42 (5%)	
Choi et al. (134)	Korea	PCR	8/123 (7%)	0/31 (0%)	16, 18, 58
Gumus et al. (135)	Turkey	PCR	37/50 (74%)	9/16 (56%)	18, 33
He et al. (136)	China	PCR	24/40 (60%)	1/20 (5%)	16
de Leon et al. (137)	Mexico	PCR	15/41 (37%)	0/43 (0%)	16, 18
Heng et al. (138)	Australia	IS PCR	8/26 (31%)	3/28 (11%)	16, 18
		PCR			
Herrara-Goepfert et al. (139)	Mexico	PCR	6/60 (10%)	7/60 (12%)	16
Mou et al. (140)	China	PCR	4/62 (6%)	0/46 (0%)	16, 18
Chang et al. (141)	China	PCR	0/48 (0%)	3/30 (10%)	6, 11
Sigaroodi et al. (142)	Iran	PCR	15/43 (35%)	1/40 (3%)	16, 18
Frega et al. (143)	Italy	PCR	9/31(29%)	0/12 (0%)	16, 18
Glenn et al. (22)	Australia	In situ PCR	25/50 (50%)	8/40 (20%)	16, 18
		PCR			
Liang et al. (144)	China	ISH	48/224 (21%)	6/37 (16%)	16, 18, 33, 58
Ahangar-Oskouee et al. (145)	Iran	PCR	22/65 (34%)	0/65 (0%)	16
Ali et al. (146)	Iraq	ISH	60/129 (47%)	3/41(7%)	16, 18, 33
Manzouri et al. (147)	Iran	PCR	10/55 (18%)	7/51 (14%)	16
Peng et al. (148)	China	PCR	2/100 (2%)	0/50 (0%)	18
Fu et al. (149)	China	PCR	25/169 (15%)	1/83 (1%)	58
		ISH			
Li et al. (150)	China	PCR	3/187 (2%)	0/92 (0%)	6, 16, 18
Wang et al. (151)	China	ISH	52/146 (36%)	3/83 (4%)	16, 18, 58
Delgado-García et al. (152)	Spain	PCR	131/251 (52%)	48/186 (26%)	16, 31, 39, 51, 59
Salman et al. (153)	United Kingdom	PCR	46/110 (42%)	1/11 (2%)	16, 31, 33, 39
Naushad et al. (34)	Pakistan	PCR	45/250 (18%)	0/15 (0%)	

*PCR, polymerase chain reaction; ISH, in situ hybridization; HPV, human papilloma virus*.

The prevalence of high-risk HPV-positive breast cancers varies between 0 or 2% in some Chinese Provinces to 86% in the US (118, 122). HPV types 16 and 18 are the most prevalent in breast cancer, however, HPV 33 and 58 are common in China and Japan (129, 130). Meta-analyses indicate that infection with high-risk HPVs is associated with an increased risk of breast cancer with an overall odds ratio of 5.4 (129, 130). However, there are marked differences between countries in the prevalence of different types of high-risk HPVs in breast cancer. The prevalence of HPV types and associated risk of breast cancer, varies between countries and regions within countries.

High-risk HPVs have been identified in the SK-BR-3 breast cancer cell line (165). High-risk HPVs have been identified in invasive breast cancers using whole-genome sequencing (166). HPV-positive koilocytes have been identified in breast cancers (167). Koilocytes are HPV infected epithelial cells with characteristic haloes surrounding a small nucleus. HPV E6 and E7 oncoproteins have been identified in breast cancer (138), however, there is a low level of transcription of these oncoproteins (168).

### Temporality

High-risk HPVs have been identified in benign breast tissues which subsequently developed HPV-positive breast cancer 1–11 years later in the same patients (166). The prior benign and later breast cancer specimens were from the same individual patients (166). Similarly, HPV-associated cervical infections can precede the development of same type HPV-positive breast cancer in the same patient (169). In a recent study based on the Taiwan National Health Insurance data base, it was shown that women with viral warts were at a 23% higher risk of developing breast cancer when compared with women not diagnosed with viral warts (170). The viral warts were assumed to be associated with HPVs. These observations confirm that HPV infections may precede the development of HPV-positive breast cancer.

### Transformation

Human papilloma viruses immortalize and transform human mammary epithelial cells (171). HPV type 16 E6 and E7 oncoproteins convert non-invasive and non-metastatic breast cancer cells to invasive and metastatic phenotypes (172).

### Transmission

Human papilloma virus-positive breast cancer is more common in young when compared with older women, an observation which is compatible with sexual transmission of HPVs among sexually active younger women (173). HPVs have been identified in peripheral blood which is a possible means by which they are transferred from infected genitals to the breast (174).

### Oncogenic Mechanisms

The prevalence of breast cancer is not increased in immunocompromised patients with AIDS or organ transplants (175). This is in contrast to the fivefold increase in HPV-associated cervical cancer in these patients (175). The implication is that any oncogenic influences of HPVs in breast cancer are indirect or there is a “hit and run” viral influence where the virus initiates cancer or plays a role in cancer development, but then disappears from tumor cells (probably by immune surveillance) by the time the cancer is clinically detected (176). HPVs appear to influence the cell cycle control enzyme APOBEC which leads to genomic instability and ultimately to breast cancer (93, 94). There is high HPV E7 oncoprotein expression in benign breast tissues and low HPV E7 expression in subsequent breast cancer which developed in the same patients (176). These observations are compatible with the viral “hit and run” hypothesis.

Human papilloma viruses may collaborate with other oncogenic viruses such as EBV and MMTV to cause breast cancer. The evidence for collaboration between HPVs and EBVs is limited and mainly consists as an association (22, 177, 178). There is preliminary experimental evidence which supports this notion (179). The influence of HPVs on MMTV and human breast cancer has been discussed in detail under the previous section on MMTV and breast cancer.

## Epstein–Barr Virus

### Identification of EBV in Human Breast Tissues

Epstein–Barr viruses are the cause of lymphomas and may be associated with additional cancers. Various EBV genes have been identified in breast cancer in a wide range of countries by *in situ* hybridization, immunohistochemistry, *in situ* PCR and by standard liquid PCR (Table 6). The details of these studies are based on a meta-analysis by Richardson et al. (180). Only case control studies with adequate controls are included in Table 6. Many studies of EBVs in breast cancer based on PCR are not valid because standard PCR techniques cannot distinguish between cancer cells and infiltrating lymphocytes. Such studies have not been included in Table 6. All listed studies used methods which identified EBV in breast cancer cells and included non-cancer breast tissue controls. Studies with negative outcomes conducted in locations where other studies had positive outcomes were excluded.

**Table 6:** Epstein–Barr virus (EBV) identification in breast cancer (case control studies).

Reference	Location	Identification technique	Breast cancer	Non cancer breast control
Labrecque et al. (154)	United Kingdom	PCR; ISH	12/19 (63%)	0/17 (0%)
Bonnet et al. (155)	France	PCR; IHC; ISH	51/100 (51%)	0/30 (0%)
Grinstein et al. (156)	US	PCR; IHC	14/33 (42%)	0/21 (0%)
Preciado et al. (157)	Argentina	PCR; IHC	24/39 (35%)	0/17 (0%)
Fawzy et al. (158)	Egypt	PCR; IHC	10/40 (25%)	0/20 (0%)
Joshi et al. (159)	India	IHC	28/51 (55%)	0/30 (0%)
Lorenzetti et al. (160)	Argentina	IHC, ISH, PCR	22/71 (31%)	0/48 (0%)
Zekri et al. (161)	Egypt/Iraq	PCR; ISH	32/90 (36%)	0/20 (0%)
Glenn et al. (22)	Australia	IS PCR PCR	5/27 (19%)	6/18 (33%)
Yahia et al. (162)	Sudan	PCR; ISH	18/18 (100%)	0/50 (0%)
El-Naby et al. (163)	Egypt	PCR; IHC	10/42 (24%)	6/42 (14%)
Pai et al. (164)	India	ISH	25/83 (30%)	0/7 (0%)

*There is a consistent identification of EBV in breast cancers when compared with mostly negative controls*.

*PCR, polymerase chain reaction; IHC, immunohistochemistry; ISH, in situ hybridization*.

The positive identification of EBV in breast cancer varies from 24 to 100% (Table 6). EBV was identified in non-cancer breast controls in only 2 of 13 studies (Table 6).

Epstein–Barr virus has been identified in over 97% of breast cancer patients and 98% of normal controls by serological methods in two independent studies conducted on Australian and Norwegian subjects (181). In a study conducted in south China, EBV antibody levels were significantly higher in breast cancer patients when compared with controls with an odds ratio of 1.7 (182).

### Epidemiology

Epstein–Barr virus is accepted as a major contributor to Hodgkin lymphoma and there are significant correlations between the incidence of both Hodgkin lymphoma and breast cancer which suggests that EBV may also contribute to some breast cancers (183). In a recent study of all subtypes of EBV in peripheral blood mononuclear cells, overall, no differences were identified between patients with breast cancer and controls (184). However, EBV subtype D was associated with an increased breast cancer risk—OR 2.86 (184).

### Temporality

Epstein–Barr virus gene sequences have been identified in benign breast tissues prior to the development of EBV-positive breast cancer (185). The prior benign and later breast cancer specimens were from the same individual patients (185).

### Transmission

Epstein–Barr virus is mostly transmitted from person to person *via* saliva. Infection is almost universal but occurs later among Western when compared with economically developing communities. Breast epithelial cells can be infected with EBV by cell to cell contact (186).

### Oncogenic Mechanisms

Epstein–Barr virus infection predisposes breast epithelial cells to malignant transformation through activation of HER2/HER3 signaling cascades (187). HER2 and HER3 are two of the cellular oncogenes known to be involved in human breast cancer development and associated with a relatively poor prognosis.

## Multiple Viruses in Breast Cancer

Epstein–Barr virus and HPV gene sequences are colocated in 32% of breast cancers can be colocated in the same women (22). Glenn et al. have demonstrated that MMTV, HPV, and EBV gene sequences are present in over 50% of Australian breast cancers and in over 20% of epithelial cells in breast milk from normal lactating women (22). In this same study, MMTV, HPV, and EBV were shown by *in situ* PCR to be located in the same breast cancer cells (22). Corbex et al. have identified EBV and HPVs in the same inflammatory breast cancers among Algerian women (174). Naushad et al. have identified EBV, high-risk HPV, and MMTV in the same breast cancers among Pakistan women (34).

The presence of EBV alongside HPV16 in cervical smears has been shown to correlate with a five to sevenfold increased likelihood of HPV integration into the host genome, suggesting that the presence of EBV may enhance genomic instability of HPV-infected cervical epithelial cells (188). EBV may also play an indirect role by interfering with the immune response to HPV-transformed cells through the production of the viral BCRF1 gene product, an interleukin-10 homolog (189). These observations provide evidence that these viruses can both collaborate to increase their oncogenic potential.

In a recent report, it has been shown that MMTV, high-risk HPVs, BLV, and EBVs may be present in normal or benign breast tissues 1–11 years before the development of breast cancer in which the same viruses have been identified (185). The prior benign and later breast cancer specimens were from the same individual patients (185). These studies were based on the same normal breast (from cosmetic surgery), benign breast and breast cancer tissues from Australian women. The investigations were conducted in laboratories in Pisa and New York (MMTV), Berkeley (BLV), and Sydney (HPV and EBV) (22, 37, 115, 166). MMTV was identified in 3 (18%) of 17 normal controls, 6 (24%) of 25 benign breast specimens and 9 (36%) of 25 breast cancer specimens. BLV was identified in 6 (35%) of 17 normal controls, 18 (78%) of 23 benign breast specimens, and 20 (91%) of 22 breast cancer specimens. High-risk HPVs were identified in 13 (72%) of 17 normal controls, 13 (76%) of 17 benign breast specimens, and 13 (76%) of 17 breast cancer specimens. EBV was not identified in any benign breast specimens but was identified in 3 (25%) of 12 breast cancer specimens. These are important observations as the presence of viruses in breast and other tissues, prior to the development of cancer, is an essential causal criterion (1).

## Discussion

Our overall aim in this review has been to evaluate the evidence in studies which employed different techniques rather than relying on one technique such as PCR. PCR based data often receives much criticism mainly because of the well recognized problems of contamination leading to false positive outcomes. This is the reason for our consideration of a range of investigative techniques and why we have been careful about which PCR data is reported in this review. We have concluded that the prevalence of MMTV, BLV, HPV, and EBV is significantly higher in breast cancer tissues when compared with normal or benign control breast tissues. The presence of these infectious agents in normal or benign tissues before the development of cancer is a key criteria for a causal role in cancer (1). MMTV, BLV, HPV, and EBV are present in normal or benign breast tissues prior to the development of the same virus invasive breast cancers in the same patients (185). In our view, each of these oncoviruses has a potential role in human breast cancer.

### Mouse Mammary Tumor Virus

With respect to MMTV and human breast cancer, the evidence is detailed and comprehensive and meets all the causal criteria except for doubts about the possible absence of an immune response. The parallels in the evidence between MMTV caused mouse mammary tumors and MMTV-like associated human breast cancer, are almost identical with the exception of the likely means of transmission. However, the underlying mechanisms for any oncogenic influences of MMTV in human breast cancer are far from clear. It is likely that any such mechanisms differ in human breast cancer from mouse mammary tumors. Despite this lack of information, it is likely that MMTV-like viruses have a similar oncogenic role in human breast cancer to MMTV in rodent mammary tumors.

However, several authors including Goedert et al., Joshi and Buehring, and Perzova et al. (4, 59, 190) have argued against a role of MMTV in human breast cancer. Goedert et al. did not identify MMTV antibodies in serum from women with proven breast cancer and therefore suggested that MMTV did not have a role in breast cancer (59). The reason for this negative finding is not clear and is in contrast to studies in which MMTV linked antibodies were repeatedly identified (56, 57). Joshi and Buehring have criticized the techniques used in several studies and have suggested the results are not reliable (4). Holland and Pogo have refuted the views of Joshi and Buehring by the presentation of published evidence (191). In our view, this evidence presented by Holland and Pogo is sound. Perzova et al. have recently suggested that the results of many studies of MMTV in breast cancer are contaminated by rodent MMTV gene sequences or by laboratory contamination (190). While such contamination may have occurred in some studies, it is unlikely to have occurred in the many independent laboratories in many countries where these studies have been conducted. In addition, it has been demonstrated in a recent study that there were no rodent MMTV sequences in any human breast cancer specimens (37). Tang and Larsson have recently reviewed studies of oncogenic viruses in a range of human cancers based on whole-genome sequencing (also known as Next Generation Sequencing or massive parallel sequencing) (192). They confirm the identification of MMTV in breast cancer but cannot exclude contamination (192, 193).

Although premature at this stage of research, it is of interest that the development of preventive and therapeutic vaccines against MMTV in humans is a practical proposition. MMTV-associated mouse mammary tumors can be prevented from developing by immunization with purified mammary tumor virus (194–196). MMTV virus envelope peptides have also been used to prevent MMTV-associated mouse breast cancers (197). The effectiveness of these vaccines is convincing evidence that MMTV is causal in MMTV-associated mouse mammary tumors. Recently, the Hochman group working in Israel, together with the Bevilacqua group in Italy, have identified the MMTV envelope protein as a target for both prevention and treatment of MMTV-associated breast and other cancers (198).

### Bovine Leukemia Virus

The possibility that BLV may have a role in human breast cancer is of great importance because of the almost universal life long consumption of beef, cow’s milk and dairy products in all Western countries and to an increasing extent in Asian countries such as Japan and China. Almost all of the evidence for such a role has been produced by Buehring et al. at The University of California at Berkeley. There is an urgent need for their work to be replicated and expanded by others.

In view of the emerging information about BLV in human breast cancer, veterinary authorities currently believe it is prudent to encourage BLV eradication programs in the dairy industry on a global basis (199).

### Human Papilloma Viruses

The evidence for a role of HPVs in breast cancer is substantial but not conclusive. Because the prevalence of breast cancer is not increased in immunocompromised patients with AIDS or organ transplants any oncogenic influences of HPVs in breast cancer are probably indirect. There may be a “hit and run” viral influence where the virus initiates cancer or plays a role in cancer development, but then disappears from tumor cells (probably by immune surveillance) by the time the cancer is clinically detected (176). HPVs also appear to influence the cell cycle control enzyme APOBEC which leads to genomic instability and ultimately to breast cancer (93, 94).

Human papilloma viruses may collaborate with other oncogenic viruses such as EBV and MMTV to cause breast cancer. The evidence for collaboration between HPVs and EBVs is limited and mainly consists as an association (22, 177, 178). There is preliminary experimental evidence which supports this notion (179). The indirect influence of HPVs on MMTV and human breast cancer has been discussed in detail under the previous section on MMTV and breast cancer.

In a recent review, Tang and Larsson state “Frequent clonal presence and expression of EBV and HPV (in breast cancers) can be ruled out, considering that transcriptomic data from more than 800 breast tumors have now been analyzed without any significant levels of these viruses being detected.” These conclusions are based on whole-genome sequencing studies published in 2013 (43). Since that time a study, using whole-genome sequencing and also based on the same TCGA series of over 800 breast tumors as the Tang et al. study, has confirmed the presence of high-risk HPVs (166). These differences in results may be because HPVs in breast cancer do not appear to express transcripts which potentially produce oncogenic proteins (193). It is possible that HPVs have an indirect oncogenic influence on breast cancer *via* their influence on APOBEC enzymes (93, 94).

### Epstein–Barr Virus

The higher prevalence of EBV in breast cancer when compared with controls is consistent. Although EBV gene sequences have been identified in benign breast tissues prior to the development of EBV-positive breast cancer, the importance of this observation is lessened because of the ubiquitous presence of EBV. Of more importance is the recognition that EBV infection predisposes breast epithelial cells to malignant transformation through activation of HER2/HER3 signaling cascades (189). The apparent collaboration between EBV and HPVs is of considerable interest but requires additional investigation (22, 177, 178). This evidence is substantial but not conclusive.

Several of these four oncoviruses are colocated in breast cancer cells and may collaborate with each other to increase their oncogenic potential. HPVs and EBV can be colocated in breast cancer cells (22). MMTV gene sequences may also be collocated with HPVs and EBVs in the same breast cancer cells (22). Because the prevalence of BLV is high in both benign breast and later breast cancer cells (78% of benign breast and 91% of breast cancer specimens), it is likely that this virus is also colocated with other virus positive benign and breast cancer cells (115). However, there is no direct evidence of such colocation. Nor is there evidence that such colocation of these viruses leads to increased oncogenic influences in breast cancer. On the other hand, in studies of women with breast cancer in Syria, it was shown that a coprevalence of EBVs and high-risk HPVs in 32% of the breast tumors was associated with high grade invasive ductal breast cancers (178). In addition there is preliminary experimental evidence that HPVs and EBVs collaborate (179). It is not known how different viruses cooperate to induce cancer.

It is possible that different viruses are associated with different types of breast cancer. For example MMTV positive human breast cancers frequently have the same histological characteristics as MMTV positive mouse mammary tumors (30, 78). These tumors are very similar to many breast ductal carcinoma *in situ* breast cancers and special breast cancer types such as medullary carcinoma, adenoid cystic carcinoma and neuro-endocrine carcinoma (78, 200, 201). In addition putative koilocytes have been observed in HPV-associated breast cancers (167).

It is important to note that the prevalence of breast cancer is not increased in immunocompromised patients with AIDS or organ transplants (175). This is in contrast to the fivefold increase in HPV-associated cervical cancer in these patients (175). The implication is that any oncogenic influences of these viruses in breast cancer are indirect or there is a “hit and run” viral influence. There is evidence in support of both notions. HPVs appear to influence the cell cycle control enzyme APOBEC which leads to genomic instability and ultimately to breast cancer (93). There is high HPV E7 oncoprotein expression in benign breast tissues and low HPV E7 expression in subsequent breast cancer which developed in the same patients (176).

### Alternative Breast Cancer Causal Hypotheses

In addition to the viral causal hypothesis for breast cancer, there are two other main hypotheses. These are (1) a genetic cause and (2) aberrant stem cells. These three hypotheses are not necessarily mutually exclusive as viruses, genetics, and stem cells could conceivably each contribute to breast cancer. It also likely that subsets breast cancers arise due to different initiation events. Thus it is unlikely there is only one cause of breast cancers and more likely it arises as a consequence of a number of events occurring.

### Genetics

During the past two decades, there has been an intense research effort directed at finding a genetic cause of breast cancer. It is clear that there is a strong genetically based susceptibility to develop breast cancer (202, 203). This disposition appears in many cases to be familial (204). The classical example is inheritance of the BRCA 1 and 2 genes. However, there is no apparent evidence that normal or mutated genes are the initial cause of breast cancer.

### Aberrant Stem Cells

The cancer stem cell theory postulates that tumors carry a subpopulation of cells that share properties of stem cells including the ability to self-renew and show indefinite replicative potential as well as initiate tumor formation, indefinite replicative potential. However, despite intense research efforts there is no apparent evidence that normal or aberrant stem cells are the initial cause of breast cancer (205, 206).

## Conclusion

The influence of oncogenic viruses is currently the major plausible hypothesis for a direct cause of human breast cancer. Genetic studies have demonstrated that there are variations in the susceptibility to develop breast cancer of which the BRCA 1 and 2 genes are the most notorious. However, although there is evidence of genetic predisposition, and cancer in general is a culmination of events, there is no evidence that inherited genetic traits are causal.

Mouse mammary tumor virus and human breast cancer—the evidence is detailed and comprehensive but cannot be regarded as conclusive.

Bovine leukemia virus and human breast cancer—the evidence is limited. However, in view of the emerging information about BLV in human breast cancer, it is prudent to encourage a BLV eradication program in the dairy industry. This has been successfully achieved by voluntary approaches in Australia and several European countries.

Human papilloma viruses and breast cancer—the evidence is substantial but not conclusive. The availability of effective preventive vaccines is a major advantage and their use should be encouraged.

Epstein–Barr virus and breast cancer—the evidence is also substantial but not conclusive. Currently, there are no practical means of either prevention or treatment.

## Author Contributions

JL, BS, and WG added to the concepts, reviewed the literature, and participated in the preparation of the manuscript.

## Conflict of Interest Statement

The authors declare that the research was conducted in the absence of any commercial or financial relationships that could be construed as a potential conflict of interest.

## Abbreviations

MMTV, mouse mammary tumor virus; BLV, bovine leukemia virus; HPV, human papilloma virus; EBV, Epstein–Barr virus; PCR, polymerase chain reaction; LTR, long terminal repeat; gag, group specific antigen; pol, polymerase; env, envelope; TCGA, the cancer genome atlas; GP, glycoprotein.
